# Paired ctDNA analysis reveals diverse resistance mechanisms to mobocertinib in EGFR exon 20 insertion NSCLC

**DOI:** 10.3389/fonc.2026.1827867

**Published:** 2026-05-19

**Authors:** Jinyong Kim, Geun-Ho Park, Sehhoon Park, Hyun Ae Jung, Se-Hoon Lee, Jin Seok Ahn, Myung-Ju Ahn, Jong-Mu Sun

**Affiliations:** 1 Division of Hematology-Oncology, Department of Medicine, Samsung Medical Center, Sungkyunkwan University School of Medicine, Seoul, Republic of Korea; 2 Department of Health Sciences and Technology, Samsung Advanced Institute for Health Science & Technology (SAIHST), Sungkyunkwan University, Seoul, Republic of Korea

**Keywords:** ctDNA, EGFR exon 20 insertion, liquid biopsy, mobocertinib, non-small cell lung cancer, resistance mechanisms, targeted therapy

## Abstract

**Background:**

Epidermal growth factor receptor (EGFR) exon 20 insertion mutations in non-small cell lung cancer (NSCLC) represent a distinct molecular subset with limited response to conventional tyrosine kinase inhibitors (TKIs). Mobocertinib, a targeted EGFR exon 20 inhibitor, shows clinical activity, yet the genomic resistance mechanisms remain poorly characterized, particularly through circulating tumor DNA (ctDNA) analysis.

**Methods:**

In this single-center prospective observational study, 22 patients with EGFR exon 20 insertion-positive NSCLC treated with mobocertinib were analyzed. Clinical outcomes were evaluated using Response Evaluation Criteria for Solid Tumors v1.1. Paired circulating tumor DNA (ctDNA) sequencing was performed pre- and post-treatment to characterize baseline mutations and acquired resistance mechanisms.

**Results:**

Mobocertinib achieved an objective response rate (ORR) of 59% and a disease control rate (DCR) of 82%, with a median progression-free survival (PFS) of 5.6 months (95% CI 3.5–9.3). Subjects who previously received amivantamab (n = 14) showed an ORR of 57.1% with median duration of response and PFS of 5.1 and 5.8 months, respectively. Insertion site variability influenced treatment efficacy, with better responses observed in helical region insertions. Baseline ctDNA absence correlated with favorable outcomes and *ATM* alterations emerged as potential negative predictive biomarkers. Resistance mechanisms were diverse, including EGFR amplification, RTK/RAS pathway alterations, and rare events such as gene fusion and small-cell lung cancer transformation.

**Conclusions:**

Mobocertinib demonstrated clinically meaningful activity, regardless of previous exposure to amivantamab, in EGFR exon 20 insertion-positive NSCLC subjects. Acquired resistance mechanisms to mobocertinib were diverse, which poses challenges to sustained efficacy, emphasizing the need for development of a tailored subsequent therapeutic strategy.

## Introduction

In non-small cell lung cancer (NSCLC), the most common actionable mutation occurs in epidermal growth factor receptor (EGFR). The development of ATP-competitive targeted therapies has revolutionized the treatment of NSCLC patients with classic EGFR mutations, such as exon 19 deletion or the L858R mutation, which represent 85–90% of all observed EGFR mutations. Based on this approach, tyrosine kinase inhibitors (TKIs) are currently the standard treatment, significantly improving survival outcomes compared with cytotoxic chemotherapy (1, 2).

However, unlike the classic EGFR exon 19 deletion or L858R mutation, the in-frame insertion mutations in exon 20 are a heterogeneous subset of EGFR mutations (3). These mutations, observed in up to 10% of EGFR mutations and 2% of all NSCLC cases, show poor response to conventional TKIs (3, 4). EGFR exon 20 insertions occur at different amino acids from D761 to C775 over the C-terminal of the C-helix or the loop following the C-helix in the EGFR tyrosine kinase domain. This results in structural changes that maintain the active state of EGFR even in the absence of ligand binding (5), thereby rendering conventional EGFR TKIs, including third-generation inhibitors, ineffective (6, 7). Consequently, these mutations exhibit an ORR of approximately 20% and a median progression-free survival (PFS) of less than 4 months.

Due to the failure to transpose conventional EGFR TKIs to exon 20 insertion treatments, novel approaches have been investigated. Amivantamab, a bispecific antibody targeting both EGFR and c-MET, plus chemotherapy showed clinical benefits and became the standard first-line treatment for NSCLC patients with EGFR exon 20 insertions, as demonstrated in the phase 3 PAPILLON trial (8). In that study, the median PFS for patients receiving amivantamab plus pemetrexed and carboplatin was 11.4 months compared with 6.7 months for chemotherapy alone (hazard ratio (HR) 0.40, 95% confidence interval (CI) 0.30–0.53).

In addition to antibodies, novel TKIs are also under development. One promising candidate is mobocertinib, an oral, first-in-class, covalent, irreversible kinase inhibitor that selectively inhibits exon 20 insertions in EGFR and HER2 kinase. The clinical efficacy of mobocertinib was analyzed in phase 1 (n = 6), phase 2 expansion (n = 22), and phase 2 extension (n = 96) studies, collectively known as the EXCLAIM study (9). Pooled analyses of these studies demonstrated an ORR of 28% and a DCR of 78%, with a median PFS of 7.3 months in patients who had previously received platinum-based treatment. These findings resulted in accelerated FDA approval of mobocertinib in September 2021. However, in October 2023, the phase 3 confirmatory study, EXCLAIM-2, failed to show an improvement in clinical efficacy over platinum-based chemotherapy, leading to the sponsor’s voluntary withdrawal of the drug (10, 11).

Due to challenges in maintaining market access for first-in-class TKIs specifically targeting EGFR exon 20 insertions, the mechanisms of resistance remain poorly understood. In the present study, an analysis was conducted using paired ctDNA-based targeted sequencing to investigate the acquired resistance mechanisms to mobocertinib and potential cross-resistance with amivantamab. We present this article in accordance with the STROBE reporting checklist.

## Methods

### Study design and participants

From January 2021 to June 2023, patients who received mobocertinib as part of an early access program at Samsung Medical Center were prospectively enrolled for analysis. All participants were treated at a single tertiary referral center in the Republic of Korea. All patients were confirmed to have EGFR exon 20 insertion mutations via droplet digital PCR (ddPCR), cell-free DNA, or tissue-based next-generation sequencing (NGS) tests. Patient demographics and treatment histories were retrieved from electronic medical records. Pre-treatment samples were collected prior to mobocertinib administration and post-treatment samples were collected upon radiologic confirmation of disease progression by the investigator.

The primary endpoint of this study was objective response rate (ORR) according to RECIST v1.1. Secondary endpoints included PFS, duration of response (DOR), overall survival (OS), and identification of baseline and acquired genomic alterations associated with clinical outcomes. Exploratory endpoints included correlation of baseline ctDNA detection status, insertion site variability, and co-mutation profiles with treatment efficacy.

### Ethical statement

The study was approved by the Institutional Review Board of Samsung Medical Center (IRB No. 2013-10-112) and conducted in accordance with the Declaration of Helsinki. Written informed consent was obtained from all participants. Clinical data were anonymized and de-identified before analysis. This study was not registered in a public clinical trial registry, as it was conducted as an observational analysis of patients treated under an early access program.

### Procedures

Mobocertinib was administered to study participants at a dose of 160 mg once daily, with the option to reduce the dose to 120 mg or 80 mg based on the treating physician’s decision. Radiologic response evaluations were conducted at intervals of at least 12 weeks. The ORR and target tumor lesions were assessed according to the Response Evaluation Criteria for Solid Tumor (RECIST) v1.1 criteria (12).

Patients with acquired resistance were defined as those exhibiting radiologic tumor progression after achieving at least stable disease (SD) as their best objective response. Blood samples were collected in EDTA tubes within 2 weeks prior to the initiation of mobocertinib and at least 2 weeks after the end of mobocertinib treatment but before the initiation of subsequent treatments.

### ctDNA extraction and analysis

Whole blood collected in EDTA tubes was centrifuged at 2,000 *g* for 10 minutes using Ficoll solution. The supernatant was transferred to a 15-mL conical tube and an additional centrifugation was performed at 2,000 *g* for 10 minutes. Plasma samples were aliquoted into 1 mL portions and stored in 1.5 mL e-tubes at −80 °C. The frozen plasma samples were shipped to Guardant Health (Guardant Health, Palo Alto, CA, USA) for DNA extraction and genomic analysis using the Guardant 360 assay (Guardant Health, Palo Alto, CA, USA; RRID: SCR_016169), which includes an 83-gene panel. All baseline and post-progression samples were analyzed using the same Guardant360 platform. The assay showed single-nucleotide variants (SNVs), insertions/deletions (indels), copy number variants, fusions, microsatellite status, and blood tumor mutation burden. For patients with samples available for real-time analysis, blood collected in Streck tubes was shipped to Guardant Health for the Guardant 360 test, which was conducted at a clinical level. The analytical performance of the Guardant360 assay has been validated, with a 95% limit of detection of ≤0.3% variant allele fraction and specificity exceeding 98% (13). No formal sensitivity analyses were conducted due to the exploratory nature and limited sample size.

### Mutation profile analysis

The baseline mutation profile was analyzed to characterize the overall mutation landscape of the study population. The correlation between baseline mutational profiles and clinical outcomes, including ORR and PFS, was also examined. For patients with available post-progression samples, acquired resistance profiles were analyzed in comparison with their corresponding baseline mutation profiles. Mutations were classified as “acquired” only when they were absent in the pre-treatment baseline sample and newly detected in the paired post-progression sample using the same Guardant360 platform. The acquired resistance mechanisms were analyzed with a focus on pathways related to known EGFR mutations.

### Statistical analysis

The data cutoff for this study was July 10, 2024. Tumor response was assessed using RECIST v1.1 criteria. The ORR was calculated as the proportion of patients who achieved a partial response (PR) or complete response (CR). The DCR was defined as the proportion of patients with CR, PR, or SD. PFS was measured from the initiation of mobocertinib treatment to the date of disease progression or death. Overall survival (OS) was defined as the time from mobocertinib initiation to the date of all-cause mortality.

Categorical variables were analyzed using the Wilson method and Fisher’s exact test. Survival analysis was performed using the Kaplan-Meier method with the log-rank test used for comparisons. HRs were calculated using the Cox proportional hazards model. All statistical tests were two-sided and a p-value less than 0.05 was considered statistically significant. No adjustment for multiple comparisons was performed due to the exploratory nature of the analyses. Data analysis was conducted using R software version 4.3.1 (R Foundation for Statistical Computing, Vienna, Austria; RRID: SCR_001905).

## Results

### Baseline demographics

Among the 23 patients treated with mobocertinib, 1 patient was withdrawn from the study due to ineligibility for mutation detection, leaving a total of 22 patients available for analysis (**Table 1**). The median age was 67 years (range 54–85 years). There were 11 male patients (50%) and 11 female patients (50%). A history of smoking, either previous or current, was observed in 8 patients (36%) and 14 patients (64%) were never-smokers. At study enrollment, the Eastern Cooperative Oncology Group Performance Status (ECOG PS) score was 1 in 19 patients (86%) and 2 in 3 patients (14%). The initial EGFR exon 20 insertion mutation was detected using ddPCR, ctDNA-based NGS, or tissue-based NGS in 7 (32%), 3 (14%), and 12 (55%) patients, respectively. Mobocertinib was administered as the second-line treatment in 6 patients (27%), third-line in 5 patients (23%), fourth-line in 4 patients (18%), and fifth-line or later in 7 patients (32%). Fourteen patients (64%) had been exposed to amivantamab prior to mobocertinib.

**Table 1:** Baseline characteristics.

Characteristic	n = 22
Median age, years (range)	67 (54–85)
Sex, No. (%)	
Female	11 (50%)
Male	11 (50)
Smoking history, No. (%)	
Never-smoker	14 (64)
Ex/Current-smoker	8 (36)
Histological subtype, No. (%)	
Adenocarcinoma	21 (95)
Squamous cell carcinoma	1 (5)
ECOG PS, No. (%)	
0	0 (0)
1	19 (86)
2	3 (14)
Median previous lines of therapy (range)	4 (2–7)
Previous amivantamab therapy, No. (%)	
Yes	14 (64)
No	8 (36)

ECOG PS, Eastern Cooperative Oncology Group Performance Status.

A total of 22 patients, comprising 22 baseline samples and 12 samples from disease progression, were used for the analysis. Genomic tests were conducted using stored plasma samples in 14 patients and real-time clinical-level Guardant tests in 19 patients. In 4 patients from the baseline sample, ctDNA was not detected, thus, insertion site analysis was not performed.

### Clinical efficacy of mobocertinib

In this study, ORR was 59%, including 13 PR patients. The DCR was 82%. Three patients showedprimary resistance and 1 patient was not available for the response evaluation. Median PFS was 5.6 months (95% CI 3.5–9.3) and median DOR was 5.8 months (95% CI 3.31–NE; **Supplementary Figures 1A, B**). Treatment outcomes did not differ based on baseline characteristics (**Supplementary Figure 2**).

### Clinical outcomes based on baseline EGFR exon 20 insertion mutation type

**Figure 1:**
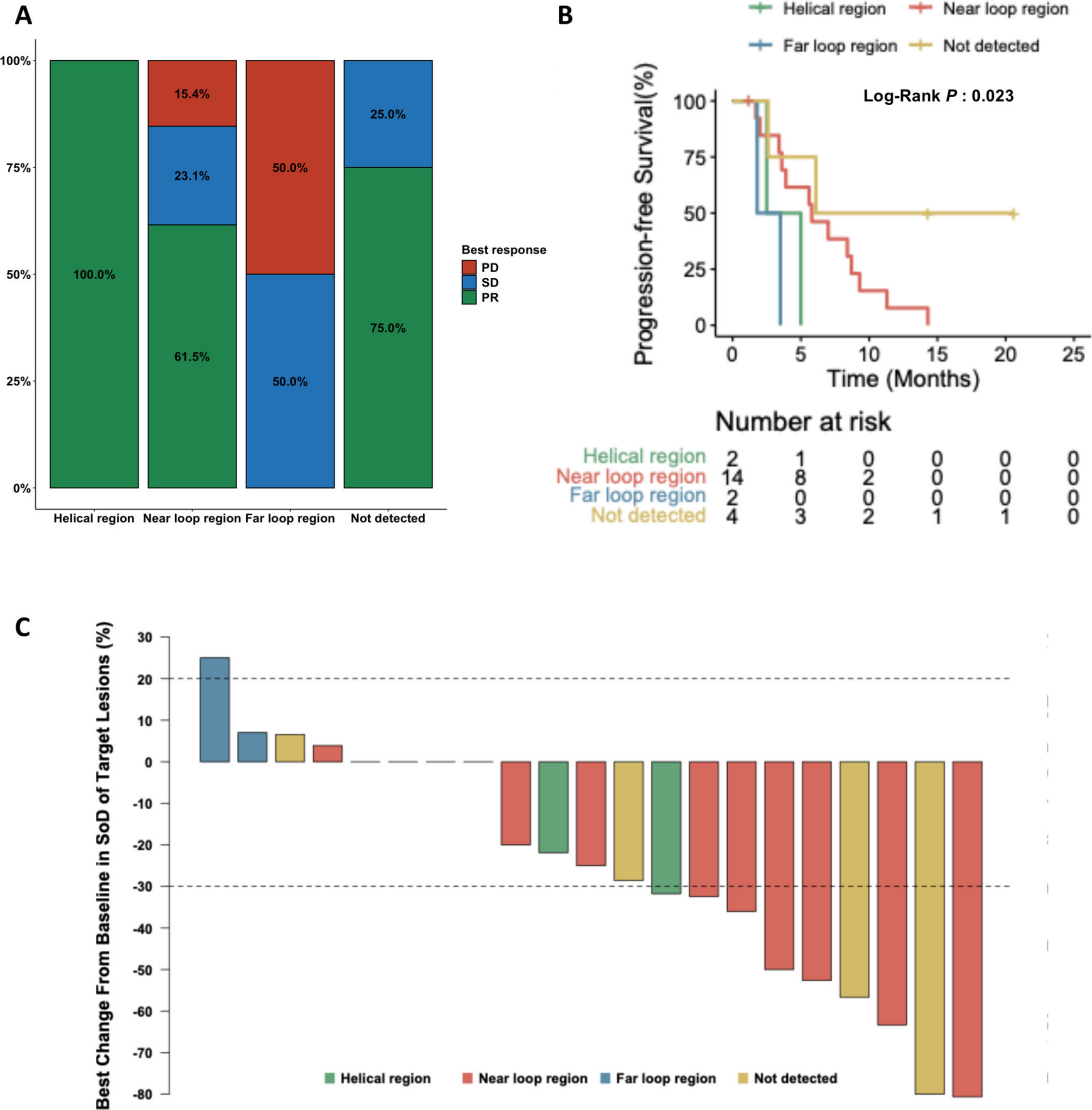
Clinical response to mobocertinib according to EGFR exon 20 insertion subtype. **(A)** Objective response rate by insertion site; **(B)** Progression-free survival stratified by insertion region; **(C)** Waterfall plot showing the maximum percentage change in target lesions.

Insertion site information detected using ctDNA-based NGS test was available for 18 patients (81.8%) in the baseline sample: insertion sites were observed at the helical region in 2 patients (11.1%), near-loop in 14 patients (77.8%), and far-loop in 2 patients (11.1%). The insertion sites of the patients who had PR as their best ORR were as follows: 100.0% in the helical region, 61.5% in the near-loop, 0% in the far-loop, and 75.0% in patients with no ctDNA detected (**Figure 1A**; **S3**).

In subgroup analysis based on the presence of baseline ctDNA, the 4 patients without EGFR mutation detected in baseline ctDNA had the longest PFS (13.4 months, 95% CI 2.6–NE). Among patients with baseline EGFR mutations, the median PFS was longest for the near-loop region (5.8 months, 95% CI: 3.6–NE), followed by helical region (3.8 months, 95% CI: 2.5–NE), and far-loop region (2.7 months, 95% CI: 1.8–NE; **Figure 1B**).

The median depth of response for treatment in all patients evaluable for target lesion was −25.0% (range −80.6% to 25.0%). The median rate of change in target volume lesions was also analyzed based on insertion site: 26.8% decrease in the helical region, 25.0% decrease in the near-loop, and 16.0% increase in the far-loop (**Figure 1C**).

### Baseline mutation profile

**Figure 2:**
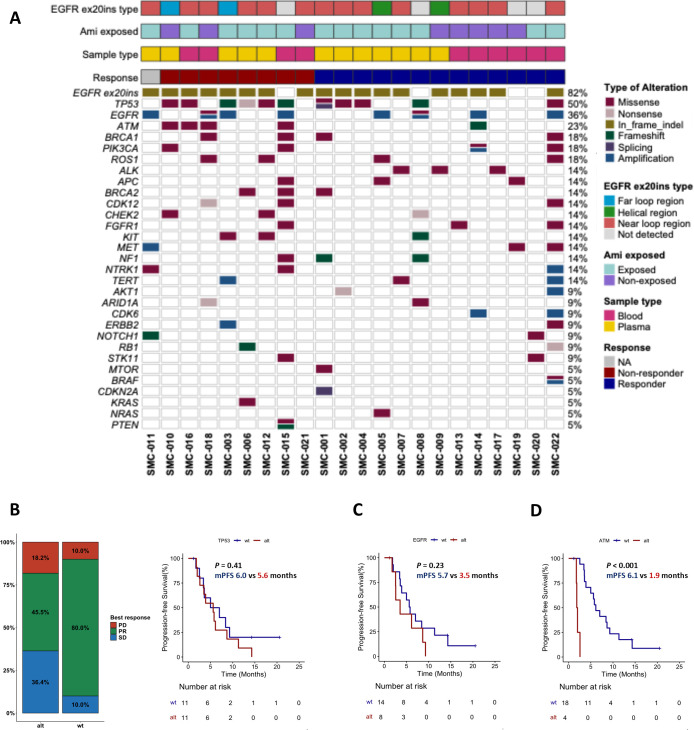
Baseline mutation profile and clinical outcome associated with mobocertinib. **(A)** Baseline mutation profile in patients with circulating tumor DNA (ctDNA) detected^†^; **(B)** Objective response rate **(ORR)** and progression-free survival (PFS) based on the presence of TP53 mutations; **(C)** PFS based on the presence of epidermal growth factor receptor (EGFR) mutation and **(D)** ATM mutation. ^†^In panel **(A)**, ‘blood’ indicates whole blood collected in Streck tubes and analyzed using clinical-grade Guardant360 testing, whereas ‘plasma’ indicates locally processed plasma samples that were centrifuged, aliquoted, and stored at −80 °C prior to shipment. Both sample types were analyzed using the same Guardant360 assay platform.

The mutation profiles from the baseline samples are shown in **Figure 2A**. The most common co-variants other than EGFR exon 20 insertion were observed in TP53 (50%), EGFR (36%), and ATM (23%). Patients with baseline TP53 mutation showed a lower response rate of 45.5% compared with patients without TP53 mutation who had a response rate of 80.0% (*P* = 0.18; **Figure 2B**). TP53 mutation did not affect the PFS between groups (*P* = 0.41). Similarly, patients with EGFR mutations or amplification (n = 8), including 2 patients with P281L and V742I co-occurring with EGFR amplification, showed no significant differences in response rate and PFS compared with the wild-type (n = 14; **Figure 2C****).**

In contrast, patients with ATM alterations demonstrated a significantly lower response rate (0% vs. 76.5%, *P* < 0.05) and shorter PFS (median PFS 1.9 months vs. 6.1 months, *P* < 0.001) than patients without alterations (**Figure 2D**).

### Resistance mechanisms

**Figure 3:**
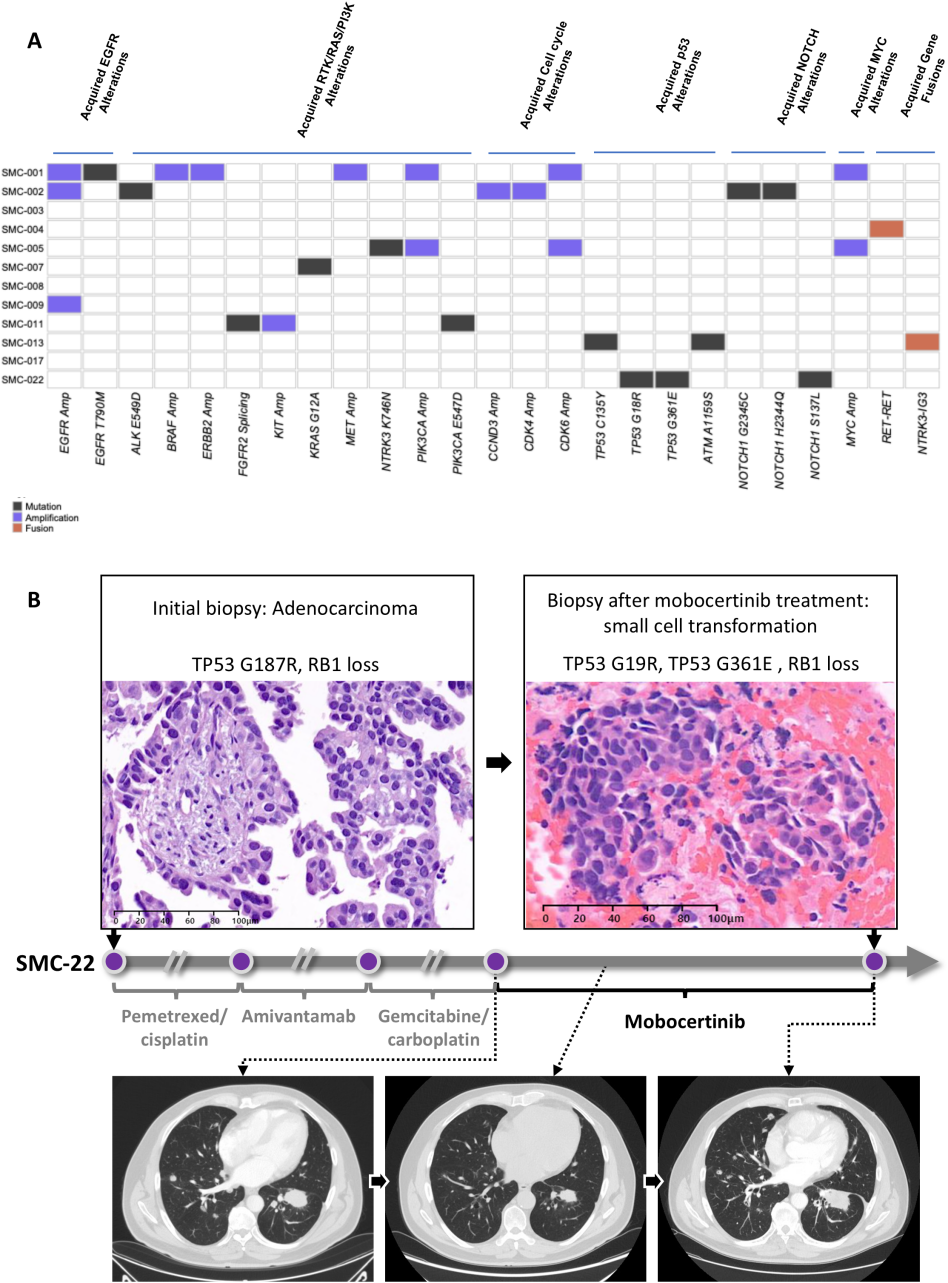
Resistance mechanism after mobocertinib. **(A)** Acquired genomic alteration identified at the time point of mobocertinib failure; **(B)** Representative case showing small cell transformation with baseline TP53 and RB1 mutation.

Acquired resistance mechanisms were analyzed in 12 patients (**Figure 3A**) and EGFR exon 20 insertion was detected in 9 patients (75.0%). Acquired resistance wasdefined as mutation newly detected after the mobocertinib treatment and the mutation was classified based on the relative pathways (**Supplementary Table 1**) (14). The most common acquired resistance mechanism was *EGFR* amplification observed in 3 patients (25.0%). One patient had acquired EGFR T790M along with *EGFR* amplification. Acquired alterations in RTK/RAS pathway were observed as ALK E549D, NTRK3 K746N, KRAS G12A, and PIK3CA E547D and amplification in *BRAF*, *ERBB2*, and *MET*. Three patients (25.0%) showed acquired genomic alteration in cell cycle-related pathways such as amplification in *CCND3*, *CDK4*, and *CDK6*. Acquired alterations in P53, NOTCH1, and MYC were observed in 2 patients. Gene fusion (RET and NTRK3 fusions) as an acquired mutation was observed in 2 patients. Loss of initial EGFR exon 20 insertion was also observed in 2 patients (SMC-004, SMC-017).

Notably, 1 case showed a small cell transformation (SMC-22) resistance mechanism. The patient was a 55-year-old, never-smoker male who was initially detected with exon 20 insertion S768_D770dup mutation. After treatment with pemetrexed with cisplatin, then amivantamab, followed by gemcitabine with carboplatin, mobocertinib was applied as fourth-line treatment. The best response to mobocertinib was PR showing maximum reduction of 50.0% in the target lesion. However, after PFS of 8.7 months, the patient showed histologic transformation to small cell lung cancer confirmed with re-biopsy (**Figure 3B**). Notably, the patient initially had TP53 G187R mutation and RB1 loss, which are characteristic of small cell lung cancer. After mobocertinib treatment, the patient lost the TP53 G187R mutation but gained TP53 G19R and G361E mutation. The RB1 loss was maintained.

### Cross resistance associated with previous amivantamab treatment

In the study population, 63.6% of the patients were previously exposed to amivantamab. The incidence of baseline TP53 mutation was higher in patients exposed to amivantamab (exposed 71.4% (10/14) vs. not exposed 12.5% (1/8), *P* < 0.05). Regarding the clinical efficacy of mobocertinib, the patients who previously received amivantamab showed an ORR of 57.1% with a median DOR of 5.6 months. Median PFS was 5.8 months, which was not significantly different from subjects not exposed to amivantamab (4.3 months, *P* = 0.86; **Supplementary Figure 4**).

## Discussion

The results of this study provided insights into the clinical outcomes and resistance mechanisms of mobocertinib in NSCLC patients harboring EGFR exon 20 insertion mutations. Due to the limited therapeutic options of this subpopulation, the findings contribute to the understanding of the efficacy of mobocertinib and the emerging resistance pathways that complicate its clinical application.

In the present study, patients showed a notable ORR of 59% and DCR of 82%, which was higher than the previously reported ORR of 28% in the pooled analyses from the EXCLAIM study (9). In contrast, median PFS was 5.6 months in this study compared with 7.3 months in the EXCLAIM trial. This discrepancy is likely due to the higher proportion of patients who received mobocertinib as fourth-line or later-line treatment (50% in this study vs. 27% in the EXCLAIM trial) (9). These findings also indicated that although mobocertinib demonstrated promising initial activity, durability remained a significant challenge. Although 64% of patients were previously treated with amivantamab, similar PFS was observed between patients with and without prior amivantamab exposure suggests a lack of cross-resistance between these agents. Both agents target EGFR exon 20 insertions. However, the distinct mechanisms between an EGFR TKI and an anti-EGFR-MET bispecific antibody likely contributed to the reduced resistance overlap as supported by real-world data (15).

Mobocertinib was designed to target the structure around the α C-helix, bind irreversibly to cysteine 797 in EGFR, and inhibit EGFR exon 20 insertion more potently (16). In the present study, the location of EGFR exon 20 insertion significantly influenced the treatment response. The response rate was highest in patients whose insertion site was in the helical region, followed by near-loop, and none in far-loop. In the EXCLAIM trial, the confirmed ORR assessed by an independent review committee was also slightly higher in patients with the near-loop insertions compared with far-loop insertions (29% vs. 25%) (9). In that study, 1 patient with C-helix insertion showed PR, similar to all patients in the present study. This observation aligned with structural analyses that near-loop insertions close to the C-helix might be susceptible to inhibition by mobocertinib due to less pronounced steric hindrance. In contrast, far-loop insertions might induce conformational changes and hinder drug binding, leading to lower efficacy. These findings highlight the need for personalized therapeutic approaches based on the specific subtypes of mutations.

The data also showed the absence of EGFR ctDNA at baseline was predictive of favorable treatment response. In the recent exploratory analysis of phase 1/2 trial of mobocertinib (NCT02716116), the detection of ctDNA EGFR exon 20 insertion in plasma before treatment was also reportedly associated with poor prognoses (17). Notably, baseline ATM alterations detected in ctDNA were associated with poor prognoses, highlighting their potential as negative predictive biomarkers. These findings indicated that ATM-driven genomic instability may contribute to resistance, warranting further studies in which targeted therapeutic strategies for this subgroup are explored. Co-mutation of TP53 mutations was prevalent at baseline (50%) and enriched in patients previously exposed to amivantamab (71.4%). In previous studies, co-mutation of EGFR exon 20 insertion and TP53 was shown associated with poor survival after treatment with platinum doublets and EGFR inhibitors (18–20). Mobocertinib treatment in patients with baseline TP53 co-mutation had a lower response rate than in subjects without co-mutation, but without a significant effect on PFS. Therefore, although TP53 mutations might indicate more aggressive disease biology, they did not necessarily predict response to mobocertinib. These observations diverged from studies in NSCLC subjects harboring classical EGFR mutations in which TP53 mutations were associated with poor outcomes (21–23). Further investigation is needed regarding exon 20 insertion mutations.

In the present study, diverse mechanisms of acquired resistance to mobocertinib were identified, reflecting the complex molecular landscape of NSCLC with EGFR exon 20 insertion. The most common resistance mechanism observed in 25% of patients was EGFR amplification and included 1 patient with concurrent T790M mutation. Other EGFR mutations in the loop following the α C-helix were also observed, indicating the tumor cells may leverage increased EGFR signaling to bypass mobocertinib inhibition. These on-target EGFR alterations are consistent with prior reports describing EGFR-dependent resistance to exon 20–targeted TKIs (24, 25). The emergence of RTK pathway alterations in KRAS, PIK3CA, and MET also indicated the alternative signaling pathways were activated as compensatory resistance mechanisms, consistent with findings from other EGFR targeted treatments (24, 26, 27).

Notably, rare, acquired resistance mechanisms such as gene fusions and small cell lung cancer transformation were also observed. Although histologic transformation was not confirmed immediately before the mobocertinib treatment, histologic transformation can be considered a resistance mechanism of mobocertinib, referring to the significant mobocertinib activity showing a PR with PFS of 8.7 months in this patient. This case emphasizes the need for biopsy upon progression because it represented a significant shift in disease biology that requires a different therapeutic approach. Small cell transformation has been documented as a resistance mechanism to osimertinib in classic EGFR mutations (28) but rarely reported in exon 20 insertion mutations, highlighting the evolving nature of resistance pathways after mobocertinib treatment.

As patients were enrolled through an early access program at a tertiary referral center, selection bias toward patients with preserved performance status may have occurred, potentially limiting generalizability. The small sample size and lack of adjustment for potential confounders may have introduced imprecision in effect estimates, particularly due to the heterogeneity in mutation subtypes and prior treatment exposures. Given the limited sample size, the study was not powered for subgroup analyses. The reliance on ctDNA-based targeted sequencing for detecting resistance mechanisms also lacked functional validation and may have missed rare genetic alterations and histological transformations. Although the Guardant360 assay demonstrates high analytical sensitivity and specificity, mutations classified as acquired resistance may still include pre-existing subclones below the detection threshold at baseline. Additionally, the lack of serial on-treatment ctDNA sampling limited assessment of the temporal dynamics of resistance mutation emergence. In addition, the short follow-up period may not have fully captured long-term resistance mechanisms and survival outcomes. Thus, larger, multicenter studies with comprehensive genomic profiling are needed to better understand and address the diverse resistance mechanisms in NSCLC patients with EGFR exon 20 insertion mutations.

In conclusion, mobocertinib demonstrated clinical activity in patients harboring EGFR exon 20 insertion mutations; however, resistance remained a significant barrier to its sustained efficacy. The findings emphasize the heterogeneity of EGFR exon 20 insertions and the need for tailored treatment approaches. Comprehensive genomic profiling is crucial for identifying the resistance mechanisms and guiding subsequent therapies, which may include combination regimens targeting multiple pathways. Further investigation is needed to elucidate the complex interplay between baseline mutations, insertion site variability, and acquired resistance to optimize the treatment strategies for the challenging population of NSCLC patients with EGFR exon 20 insertion.

## Conclusions

Mobocertinib demonstrated clinically meaningful activity in patients with EGFR exon 20 insertion–positive NSCLC, including those previously treated with amivantamab, suggesting limited cross-resistance between these agents. Baseline ctDNA status and ATM alterations may serve as predictive biomarkers of response. Acquired resistance mechanisms were heterogeneous, involving EGFR amplification, bypass pathway activation, gene fusions, and histologic transformation. These findings underscore the importance of comprehensive molecular monitoring to inform personalized therapeutic strategies in this challenging molecular subtype. Although mobocertinib is no longer commercially available, understanding resistance mechanisms remains clinically relevant for the development of next-generation exon 20-targeted agents.

## Data Availability

The raw data supporting the conclusions of this article will be made available by the authors, without undue reservation.

## Supplementary material

Supplementary Figure 1 (A) Overall median progression-free survival (PFS) and (B) duration of response.

Supplementary Figure 2 Subgroup analysis based on baseline characteristics.

Supplementary Figure 3 Swim-lane plot indicating the duration of response to mobocertinib.

Supplementary Figure 4 Progression-free survival (PFS) based on exposure to amivantamab in the study population.

Supplementary Table 1 Acquired resistance mutations and signaling pathways
