# Supplementary material for: Paired ctDNA analysis reveals diverse resistance mechanisms to mobocertinib in EGFR exon 20 insertion NSCLC
Source: Front Oncol. 2026 May 19;16:1827867. doi: 10.3389/fonc.2026.1827867 (PMC13225986; doi:10.3389/fonc.2026.1827867)
Supplement: Supplementary Figure 1 — (A) Overall median progression-free survival (PFS) and (B) duration of response. [file Presentation1.pptx]

## Slide 1
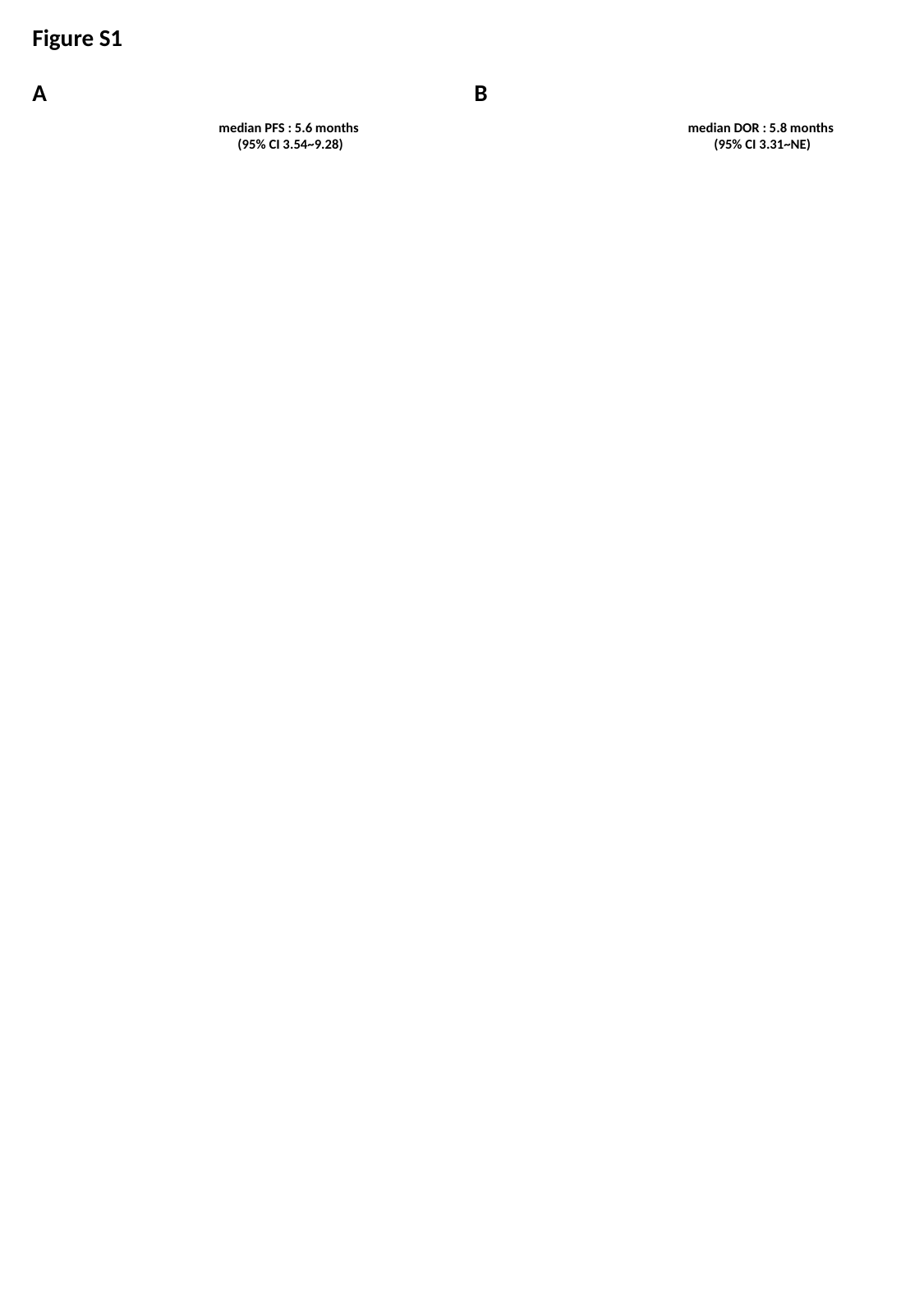

Figure S1
A
B
median PFS : 5.6 months
(95% CI 3.54~9.28)
median DOR : 5.8 months
(95% CI 3.31~NE)

## Slide 2
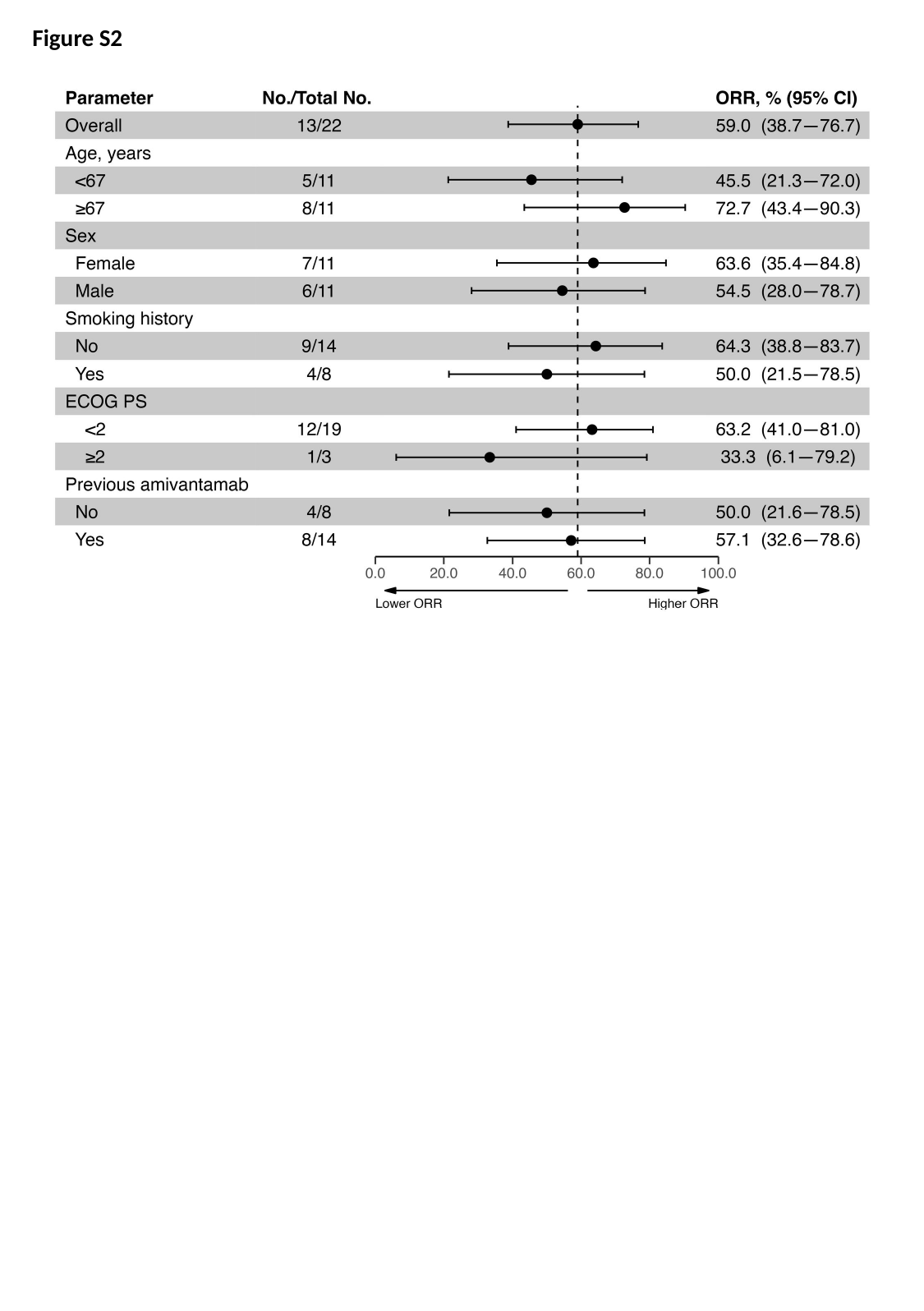

Figure S2

## Slide 3
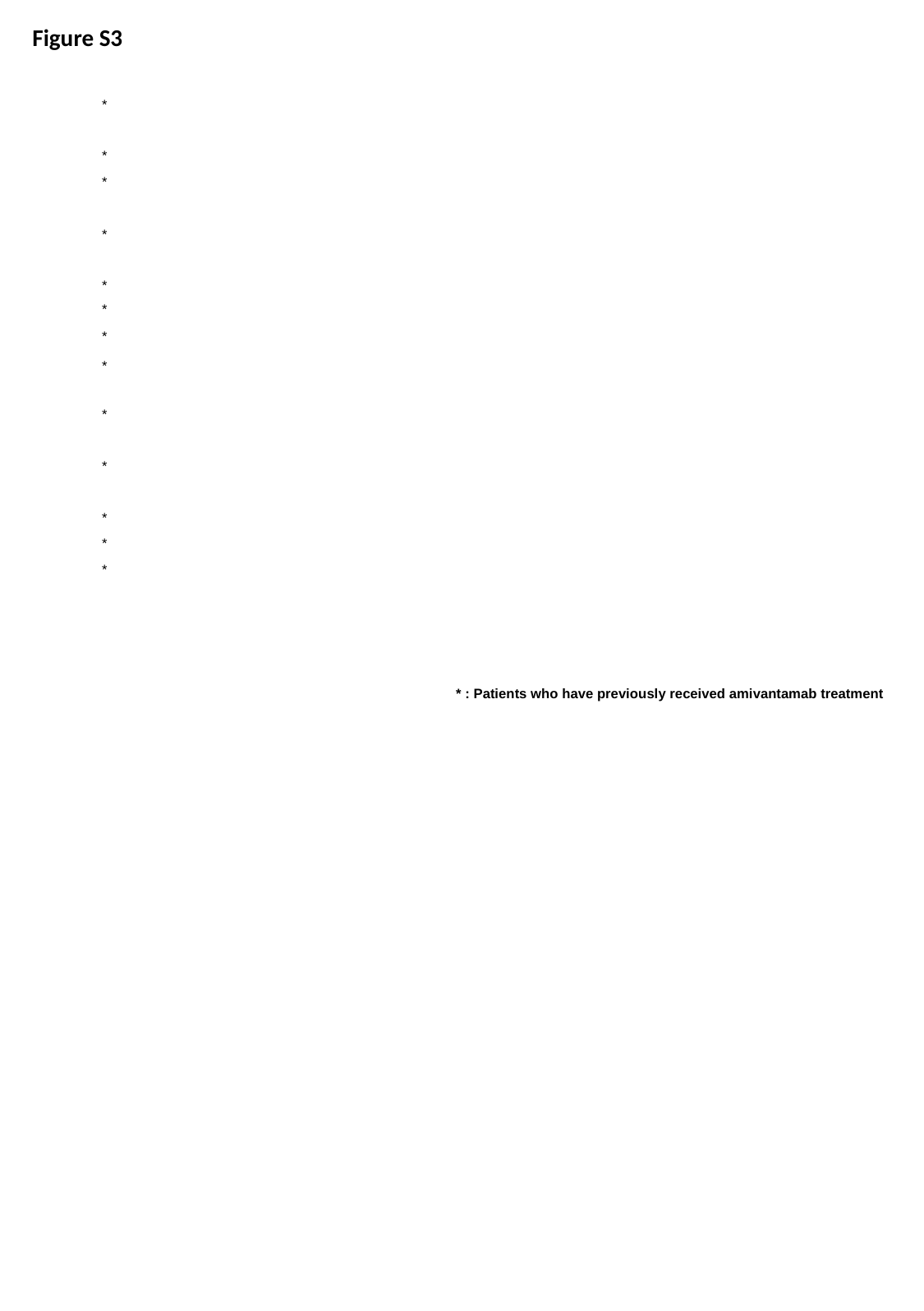

Figure S3
*
*
*
*
*
*
*
*
*
*
*
*
*
* : Patients who have previously received amivantamab treatment

## Slide 4
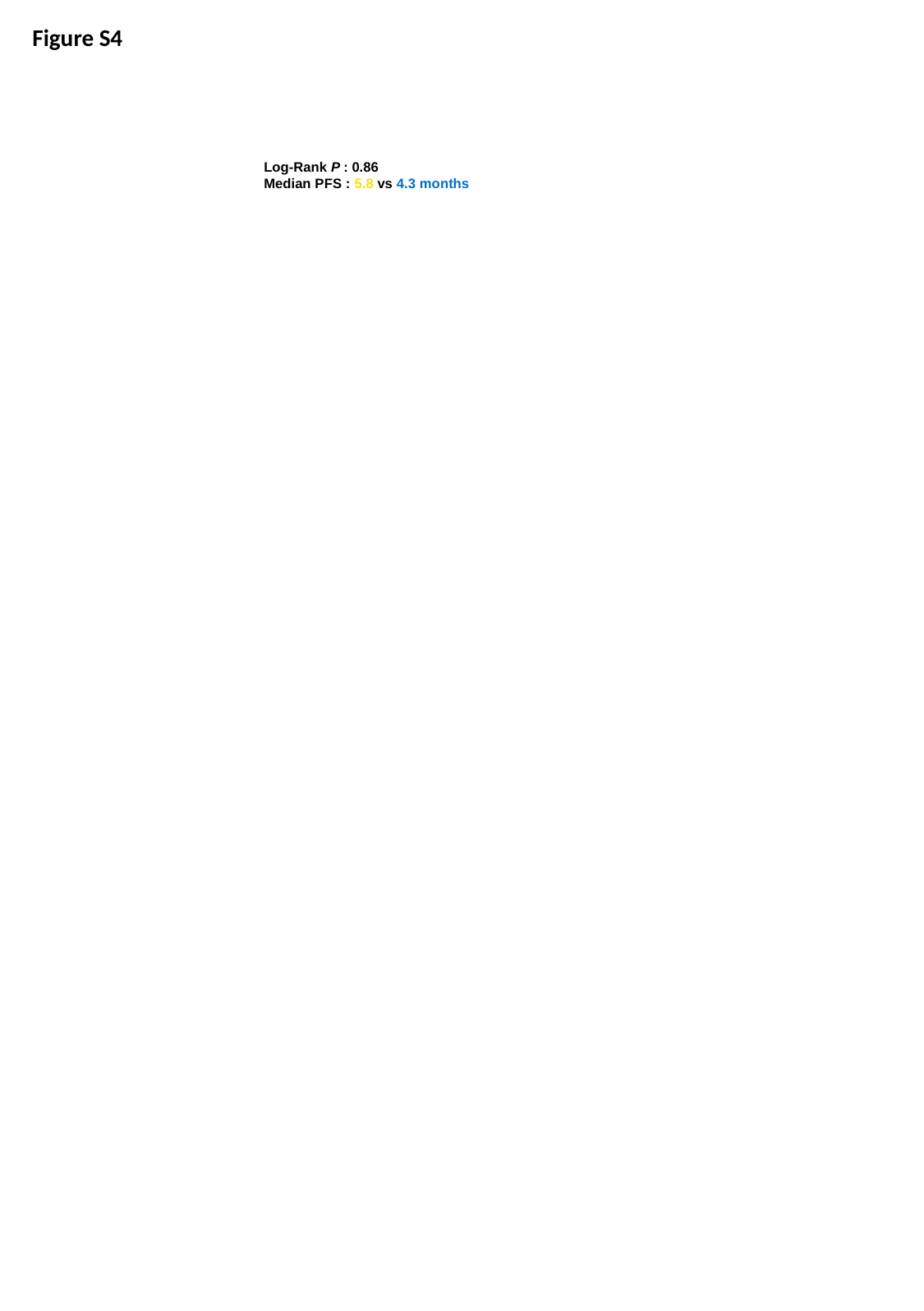

Figure S4
Log-Rank P : 0.86
Median PFS : 5.8 vs 4.3 months
